# Should natalizumab be used despite JC virus positivity?—Commentary

**DOI:** 10.1177/13524585251346378

**Published:** 2025-05-30

**Authors:** Anna Karin Hedström

**Affiliations:** Department of clinical neuroscience, Karolinska Institutet, Stockholm, Sweden

Natalizumab has long held a central place among the most effective therapies for relapsing-remitting multiple sclerosis (MS), offering rapid and sustained suppression of inflammatory disease activity. Its high efficacy has made it particularly valuable in the management of patients with aggressive disease, often producing marked reductions in relapse rates and radiological activity within months.^1^ However, its association with progressive multifocal leukoencephalopathy (PML) in JC virus (JCV)-seropositive individuals led to its temporary withdrawal shortly after approval and has remained a source of concern. Although the drug was reintroduced with strict risk stratification protocols,^2^ the emergence of alternative high-efficacy therapies has made its continued use in JCV-seropositive patients increasingly debated.

In the current journal issue, William Carroll makes a strong case for the selective use of natalizumab in JCV-seropositive patients under well-defined conditions. He emphasizes the accumulating evidence supporting extended interval dosing (EID), which appears to preserve therapeutic efficacy while substantially reducing the risk of PML.^3^ Observational and trial data suggest that in carefully selected individuals, particularly those without prior immunosuppressant exposure and with low to moderate JCV antibody indices, a limited course of natalizumab can be administered with an acceptable safety profile,^4^ allowing time to control disease activity and plan long-term treatment strategies.

Conversely, Gloria Dalla Costa and co-authors present a precautionary approach grounded in the unpredictability of PML onset, limitations in current risk prediction tools, and the clinical challenges associated with natalizumab withdrawal, including the risk of rebound disease.^5^ They argue that with the availability of equally effective alternatives, such as anti-CD20 agents or cladribine, the risks inherent to natalizumab use in JCV-positive individuals are no longer justified.

What emerges from both perspectives is a shared recognition that risk can be reduced but not eliminated. Even with EID, uncertainty remains. Moreover, the need for sustained monitoring, the psychological burden of potential PML, and the complexities of treatment cessation raise significant concern even in short-term use. In the absence of reliable predictive biomarkers for PML, a cautious and personalized approach remains warranted. For a small subset of patients with highly active disease unresponsive to other therapies, a short treatment course under close monitoring may still be justified. For the majority of JCV-positive individuals, particularly those with access to alternative treatment options, the case for avoiding natalizumab is persuasive.

Ultimately, the controversy highlights the increasing complexity of treatment decisions in MS, as clinicians weigh potent therapeutic options against evolving risk profiles and a growing emphasis on individual patient priorities. Future findings from large-scale observational studies and ongoing biomarker research may help clarify when, and for whom, natalizumab can be used safely in the context of JCV seropositivity. Until then, clinical equipoise persists.
